# Supplementary material for: Single cell analyses reveal contrasting life strategies of the two main nitrifiers in the ocean
Source: Nat Commun. 2020 Feb 7;11:767. doi: 10.1038/s41467-020-14542-3 (PMC7005884; doi:10.1038/s41467-020-14542-3)
Supplement: Supplementary file 1 — Supplementary Information [file 41467_2020_14542_MOESM1_ESM.pdf]

Single cell analyses reveal contrasting life strategies of the two main nitrifiers in the ocean

Kitzinger et al.

Supplementary Information: Single cell analyses reveal contrasting life strategies of the two main nitrifiers in the ocean

Supplementary Methods

Nitrospinae probe design and CARD-FISH conditions

As the previously available probes for Nitrospinae[1,2] did not target all Nitrospinae OTUs identified in the present study, and only a subset of the deposited Nitrospinae 16S rRNA gene sequences (Supplementary Table 7), a new catalyzed reporter deposition (CARD-) FISH probe targeting the Nitrospinaceae, the family containing all known nitrite oxidizing Nitrospinae, was developed. Nitrospinae 16S rRNA gene amplicon sequences obtained in this study were aligned to the SILVA SSU Ref NR 99 128 alignment[3] in Arb version 6.1. using the SINA aligner[4]. To ensure optimal coverage of all Nitrospinae OTUs found in the present study, only sequence positions amplified by the used primer pair[5] were considered for probe design. A probe (Ntspn759, 5' CCCTGGCTTTCGTATCT 3') was designed using the probe design tool implemented in ARB, further manually refined and evaluated *in silico* using MathFISH[6]. Competitor probes to non-target organisms with single mismatches were designed manually (Ntspn759_comp1, 5' CCCTGGCTTTCGTACCT 3' and Ntspn759_comp2, 5' CCCTGGCTTTCGCATCT 3') and included in all CARD-FISH experiments. The newly designed probe Ntspn759 targets 91% (263 of 288) Nitrospinae sequences included in SILVA SSU Ref NR 99 128, for comparison, probe Ntspn693[1] and Ntspn-Mod[2] target only 15% and 79%, respectively (Supplementary Table 7).

Optimal formamide concentration[7] for probe Ntspn759 hybridization experiments was evaluated for CARD-FISH on a *Nitrospina gracilis* pure culture[8] and confirmed on samples from the GoM. Optimal formamide concentration was 15% at 46°C hybridization temperature (Supplementary Figure 14).

Nitrospinae abundances were determined by CARD FISH following Pernthaler et al.[9]. Briefly, cells were immobilized on the filters by embedding in 0.2% low gelling agarose and endogenous peroxidases were inactivated by incubation in 0.01 M HCl for 10 min. Cells were permeabilized by HCl

permeabilization (0.1 M HCl for 1 min) and lysozyme digestion (10 mg ml^{-1} in 50 mM EDTA and 100 mM Tris-HCl at 37°C for 1 h). Filters were hybridized with horseradish peroxidase labeled oligonucleotide probes at 46°C for up to 3.5 h. Signal amplification was done with OregonGreen labeled tyramides at 48°C for 15 to 30 min. Before enumeration on an epifluorescence microscope (Axioplan 2, Zeiss), cells were counterstained with DAPI. For each CARD-FISH experiment, negative controls with a peroxidase linked probe NonEUB[10] were done to exclude non-specific binding of oligonucleotides or insufficient inactivation of endogenous peroxidases.

Analysis of $^{15}N/(^{14}N + ^{15}N)$ ratio variability in Nitrospinae nanoSIMS measurements

We assessed whether sufficient Nitrospinae cells have been measured by nanoSIMS following Svedén et al.[11]. Briefly, we calculated the mean and standard error for randomly subsampled Nitrospinae ROIs, where the error of randomly subsampled ROIs of one population should be < 10% to indicate that sufficient cells have been analyzed. Our analysis showed that the standard error for Nitrospinae $^{15}N/(^{14}N+^{15}N)$ values was <10% after measurement of 3, 2 and 3 cells in the ^{15}N-ammonium, ^{15}N-urea and ^{15}N-nitrite treatments, while for the ^{15}N-cyanate treatment, one Nitrospinae cell with higher activity caused the error to remain 19% after analysis of all 19 Nitrospinae cells (Supplementary Figure 13). For non-Nitrospinae cells, the standard error of $^{15}N/(^{14}N+^{15}N)$ values was <10% after measurement of 74, 60, and 185 cells in the ^{15}N-ammonium, ^{15}N-cyanate and ^{15}N-nitrite treatments (Supplementary Figure 13). For ^{15}N-urea, the standard error did not drop below 10% even after analysis of 140 non-Nitrospinae cells, likely due to the high metabolic variability, in this group, where only some are able to use urea (Supplementary Figure 13, [12]).

DNA and RNA analyses

Nucleic acid sampling and extraction

1L seawater each was filtered in replicates onto 0.22 µm cartridge filters (Sterivex™, Millipore), filled with lysis buffer (50 mM Tris-HCl, 40 mM EDTA, 0.73 M sucrose) for DNA analyses or RNA stabilizing buffer (25 mM sodium citrate, 10 mM EDTA, 5.3 M ammonium sulfate, pH 5.2) for RNA analyses.

Filters were stored at -20°C or flash frozen in liquid nitrogen, for DNA and RNA samples respectively. DNA was extracted using a phenol:chloroform protocol[13]. Cells were lysed by adding lysozyme (2 mg in 50 µl of lysis buffer per filter) directly to the cartridges, sealing the cartridges, and incubating for 45 min at 37°C. Proteinase K (1 mg in 100 µL lysis buffer, 100 µl 20% SDS) was added, and the cartridges were resealed and incubated for 2 hours at 55°C. The lysate was removed, and DNA was extracted once with phenol:chloroform:isoamyl alcohol (25:24:1), once with chloroform:isoamyl alcohol (24:1) and then concentrated by spin dialysis using Ultra-4 (100 kDa, Amicon) centrifugal filters.

RNA was extracted using a modification of the *mir*Vana™ miRNA Isolation kit (Ambion)[14]. Cartridges were thawed on ice, RNA stabilizing buffer was then expelled and discarded, and cells were lysed by adding Lysis buffer and miRNA Homogenate Additive (Ambion) directly to the cartridges. Following vortexing and incubation on ice (10 min), lysates were transferred to RNAase-free tubes and processed through an acid-phenol:chloroform extraction according to the kit protocol. The TURBO DNA-free™ kit (Ambion) was used to remove DNA, and the extract was purified using the RNeasy MinElute Cleanup Kit (Qiagen).

16S rRNA gene sequencing and analysis

Relative abundances of microorganisms were assessed via 16S rRNA gene amplicon sequencing. Amplicons were generated by PCR using equal amounts of DNA template (1 ng), Platinum® PCR SuperMix (Life Technologies), and primers F515 and R806 encompassing the V4 region of the 16S rRNA gene[5]. Both forward and reverse primers were barcoded and appended with Illumina-specific adapters. Thermal cycling involved: denaturation at 94°C (3 min), followed by 30 cycles of denaturation at 94°C (45 sec), primer annealing at 55°C (45 sec) and primer extension at 72°C (90 sec), followed by extension at 72°C for 10 min. Amplicons were analyzed by gel electrophoresis to verify size (~400 bp) and purified using Diffinity RapidTip2 pipette tips (Diffinity Genomics, NY). Amplicons from different samples were pooled at equal concentrations and sequenced on the Illumina MiSeq Platform using a Reagent Kit V2 (500-cycles) and a Nano Flow Cell.

Barcoded sequences were de-multiplexed, trimmed (length cutoff 100 nt), and filtered to remove low quality reads (average Phred score < 25) using Trim Galore (http://www.bioinformatics.babraham.ac.uk/projects/trim_galore/). Paired-end reads were merged using FLASH[15], with a minimum average length of 250 nt for each read, minimum average length of 300 nt for paired read fragments, and maximum allowable fragment standard deviation of 30 nt. The number of trimmed and merged reads per sample ranged from 11,842 – 21,970. Chimeric sequences were detected by reference-based searches using USEARCH[16] and removed from the sequence pools. Operational Taxonomic Units (OTUs) were defined by clustering at 97% sequence identity using open-reference picking with the UCLUST algorithm[16] in QIIME1[17]. The average number of sequences assigned per OTU was 836 (range 646 – 1,138). Taxonomy was assigned to OTUs using the Greengenes database[18]. Singleton sequences and sequences affiliated with mitochondria and chloroplast were removed from any further analysis. Proportional abundances of orders constituting >0.5% of the community were calculated after rarefaction based on the sample with the lowest number of reads (11,842 reads).

Metagenome sequencing and Nitrospinae metagenome assembled genome reconstruction

Metagenomic libraries were constructed as previously described[12], using the NEBNext® UltraTM II FS DNA Library Prep Kit for Illumina, creating inserts with an average fragment sizes of 550 bp. Samples were sequenced on one lane of an Illumina HiSeq2500 flow cell in Rapid Run Mode using a using 2 × 250 bp cycle kit at Georgia Tech's High-Throughput DNA Sequencing core facility.

bbduk (BBMap v. 36.32 - Bushnell B. - sourceforge.net/projects/bbmap/) was used to remove adapters and residual phiX sequences, and to further quality-filter (ktrim=r k=21 mink=11 hdist=2 minlen=149 qtrim=r trimq=15) the paired-end Illumina reads. Quality-filtered reads were assembled with Metaspades v. 3.10.1 (-k 21,33,55,77,99,127)[19]. Each individual read set was mapped against each assembly to assist in differential-coverage genome binning using BBMap v. 36.32 (BBMap - Bushnell B. - sourceforge.net/projects/bbmap/). Large (>2 kb) scaffolds were clustered into Metagenome-Assembled Genomes (MAGs) by oligonucleotide frequency (k=4) and read coverage

using Metabat2 v. 2.12.1[20]. MAGs were dereplicated using dRep v. 1.4.3[21] (completeness >40%, contamination <10%, ANI >99% and genome size >200kb). MAGs were further clustered into groups (referred to as Nitrospinae population clusters A, B, C) based on average nucleotide identity (95% ANI) using gANI-MiSI [22] and hclust(method="complete") in R v. 3.5.1[23].

Nitrospinae MAGs were identified using GTDB-Tk v. 0.2.2 (https://github.com/Ecogenomics/GtdbTk) with database release 86, which is based on the Genome Taxonomy Database[24]. MAGs were reassembled/ polished by iteratively mapping reads to dereplicated metagenome bins (contigs >2kb) using an automated version of the method used in[25–27]. Briefly, BBMap v. 36.32 was used to map reads (>98% identity) and SPAdes v. 3.10.1[28] was used to reassemble mapped reads using the previously binned contigs as “trusted-contigs”. Initially, the reassembly ran for ten rounds and was repeated (in blocks of ten rounds) until reassembly became self-consistent (based on genome size, completeness, contamination and number of scaffolds as evaluated with CheckM v. 1.0.7). Self-consistency was established by evaluating correlation between CheckM-calculated statistics and the number of rounds over ten rounds of reassembly in R v. 3.5.1 [23] with cor.test() for each statistic. If all statistics were not correlated with round number (p-values for correlation >0.05), the bin was considered to be self-consistent. Between each round of reassembly, reassembled contigs were filtered based on consistency in mapping depth, consistency in tetranucleotide composition and length (> 2kb). Mapping depth and tetranucleotide composition was evaluated in R v. 3.5.1 [23] through the construction of null models using 2 kb subsequences from the newly reassembled genome. A normally distributed null model for mapping depth was constructed using the mean and standard deviation of median depth for the 2 kb subsequences. P-values were calculated for reassembled contigs based on the null model of mapping depth with pnorm() and corrected for multiple testing using p.adjust(method=”BH”). Contigs were rejected if the adjusted p-value < 0.05. A multidimensional null normal distribution for tetranucleotide frequency was constructed by conducting a principle coordinate analysis (PCoA) of log(tetranucleotide counts+1) for 2 kb subsequences and full reassembled contigs. This procedure predicts weighted normalized variance (as a distance from the origin) for each tetranucleotide pattern. P-values were calculated for

reassembled contigs based on the null model of tetranucleotide patterns with pnorm() and corrected for multiple testing using p.adjust(method="BH") in R with a FDR of 0.05. Contigs were rejected if the adjusted p-value < 0.05. Metagenome sequencing statistics and information on dereplicated Nitrospinae MAGs are listed in Supplementary Tables 5 and 1, respectively.

Single-gene phylogenetic reconstruction

16S rRNA gene sequences from metagenomes were identified in metagenomic assemblies with nhmmer (hmmer v. 3.1b2)[29] using rfam models[30] for small subunit rRNAs (RFAM: RF00177, RF01959, RF01960). Minimum overlap between sequence and model was 300 nucleotides. Sequences were classified using the RDPclassifer[31] as implemented in Mothur v. 1.39.5 [32].

Gene predictions for each metagenomic assembly were made using Prodigal[33], using the metagenome option (-p meta). Marker genes of nitrite, urea, and cyanate utilization, as well as RNA polymerase genes were extracted from metagenomic assemblies: nitrite oxidoreductase subunit alpha (*nxrA*), urease subunit alpha (*ureC*), cyanate lyase/hydratase (*cynS*), and the β subunit of bacterial RNA polymerase (*rpoB*), respectively. hmmsearch (hmmer v. 3.1b2)[34] was used to identify genes of interest (*nxrA* (PF00384.17), *ureC* (PF00449.15), *cynS* (PF02560.14), *rpoB* (PF00562, RNA_pol_Rpb2_6)), with the requirement that the protein sequence and hmm model align over at least 70% of the length of the model and that the reverse search of the identified protein sequence against the pfam database returned the target model as the best hit.

Reference databases were constructed for *nxrA, ureC*, *cynS* and *rpoB* by screening amino acid sequences encoded by all genomes publicly available within the International Nucleotide Sequence Database Collaboration[35] and genomes classified as *Nitrospina* within the Integrated Microbial Genomes & Microbiomes system v.5.0 [36]. Screening of reference genomes was done using annotated amino acid sequences, where available, and were based on amino acid sequences predicted by Prodigal otherwise. Genes of interest were identified using hmmsearch (hmmer v. 3.1b2) using the same models and criteria as were used for screening the metagenomic assemblies.

Metagenomic-encoded sequences were added to the reference databases and aligned with mafft-linsi v. 7.397 [37] and trimmed using trimal v. 1.4.1 (parameter: -automated1)[38]. Phylogenetic reconstruction was calculated with IQ-TREE v. 1.6.2 [39] with automated model selection under models automatically determined to be the best-fit model by ModelFinder[40]. Confidence was assessed with 1,000 ultrafast bootstraps[41]. Resulting trees were used to manually define clades in which Nitrospinae sequences belonged to. Sequences for Nitrospinae-related clades were extracted, realigned and trees were calculated as above. These final trees were visualized using ITOL[42].

Unassembled metagenomic and metatranscriptomic reads were used to quantify *ureC*, *cynS*, *nxrA* and *rpoB* in these datasets. mRNA reads were screened by BLASTX v. 2.8.1+ against the dataset assembled for phylogenetic analysis (see above). Positive BLASTX matches were defined by an e-value $<10^{-6}$, bit score ≥ 50 and alignment length ≥30 amino acids. Reads were added to alignments used for calculating phylogeny of each gene of interest using the --add-fragments option in mafft v. 7.397 and placed into single gene trees using the evolutionary placement algorithm (RAxML v. 8.2.11)[43]. Reads placed into the tree with pendant length >0.1 were ignored. The number of reads placed onto individual branches was inferred from integrating classification likelihoods for each branch/read combination. Fragments per kilobase per million reads (FPKM) values were calculated based on the number of inferred read pairs for which one or both reads placed into a specified location in the tree, divided by the median gene length in the reference alignment (in kb) divided by the number of total metagenomic read pairs or ribosomal-RNA free metatranscriptomic read pairs (in millions). Median gene length for calculation of FPKM values was 3,435 nt for *nxrA,* 1,704 nt for *ureC*, 450 nt for *cynS* and 4,205 nt for *rpoB*.

Supplementary Tables

Supplementary Table 1 Nitrospinae metagenome assembled genome (MAG) information and relative MAG abundances. Completeness, contamination and strain heterogeneity were calculated using CheckM v. 1.0.7. Abundances per sample are given as percent metagenomic fragments mapping to the respective MAGs compared to the total number of fragments per metagenome. Note that these mappings were done non-competitively for each MAG. Therefore, average population cluster abundances were used for calculating their abundance, as MAGs of one population cluster represent different strains with very similar genome content. For calculation of the abundance of population cluster A, only MAG 32A and 76A were considered, as the binned genome size of 304A was smaller, which resulted in an aberrantly low number of mapped reads to this MAG. Note that the abundance of MAG 36C may be an underestimate due to its low completeness.

MAG ID	MAG Cluster	Scaffold length (MB)	Completeness (%)	Contamination (%)	Strain heterogeneity (%)	Binned 16S	Binned nxrA	Binned ureC	Binned cynS	Station 1, 18.0 m (%)	Station 2, 12.0 m (%)	Station 2, 14.0 m (%)	Station 2, 16.5 m (%)	Station 3, 14.0 m (%)
GoM_MAG_304A	A	1.894	79.62	2.62	0					0.258	0.003	0.016	0.036	0.165
GoM_MAG_32A	A	2.546	84.62	2.3	16.67	X	X			0.352	0.003	0.021	0.043	0.229
GoM_MAG_76A	A	2.621	93.37	2.56	0	X	X	X		0.364	0.003	0.021	0.044	0.243
GoM_MAG_55B	B	2.639	92.49	5.13	0	X	X		X	0.134	0.008	0.046	0.101	0.155
GoM_MAG_90B	B	2.653	94.82	4.27	0	X	X	X		0.134	0.008	0.047	0.103	0.154
GoM_MAG_111B	B	2.529	91.4	2.71	0	X	X			0.132	0.008	0.044	0.097	0.148
GoM_MAG_36C	C	1.926	48.1	2.14	0			X		0.050	0.003	0.008	0.011	0.043
Total percent of fragments mapping to Nitrospinae MAGs										0.530	0.016	0.090	0.193	0.443

Supplementary Table 2 Overview of key genes manually annotated in GoM Nitrospinae MAGs. Green cell shading indicates presence of all gene subunits (for multi-subunit enzymes), orange presence of some subunits.

	MAG Cluster A			MAG Cluster B			MAG Cluster C
**Gene**	**GoM_MAG_32A**	**GoM_MAG_76A**	**GoM_MAG_304A**	**GoM_MAG_55B**	**GoM_MAG_90B**	**GoM_MAG_111B**	**GoM_MAG_36C**
Nitrite oxidoreductase (NxrABC)							
Nitrite reductase, copper containing (NirK)							
Ferredoxin nitrite reductase, assimilatory (NirA)							
Nitrite transporter (NirC)							
Nitrate/nitrite transporter (NrtP)							
Nitrate ABC transporter							
Ammonium transporter (AmtB)							
Urea ABC transporter (UrtABCDE)							
Urease accessory proteins (UreDEFG)							
Urease (UreABC)							
Cyanase (CynS)							
Spermidine ABC transporter							
Amino acid ABC transporter							
(Oligo-) Peptide ABC transporter							
Sugar transporter SemiSWEET							
C4-dicarboxylate ABC transporter (fumarate, malate, succinate, oxaloacetate)							
Hydrogenase, NiFe 3b hydrogenase							
Chlorite dismutase (Cld)							
ATP citrate lyase (rTCA cycle)							
2-oxoglutarate:ferredoxin oxidoredutase (rTCA cycle)							

Supplementary Table 3 Gibbs free energy for ammonia oxidation and nitrite oxidation calculated for standard conditions and for conditions in the GoM, i.e. 28°C, salinity 34.8 ppt, pH 7.8, NH_4^+ 0.330 µM, NO_2^- 0.85 µM, NO_3^- 2.25 µM, O_2 10 µM. NH_3 concentration (0.01 µM) was calculated from NH_4^+ concentrations following Beman et al.[44] as $NH_3 = NH_4^+ * 10^{(pH-pKa)}$ using pH 7.8 and pKa 9.3.

**Process**	**Reaction**	**ΔG⁰**	**ΔG′⁰**
Ammonia Oxidation	$NH_3 + 1.5O_2 \rightarrow NO_2^- + H_2O + H^+$	-271.8	-262.4
Nitrite Oxidation	$NO_2^- + 0.5O_2 \rightarrow NO_3^-$	-82.1	-65.3

Supplementary Table 4 $^{15}N^{13}C$-tracers and ^{14}N-pools added for assimilation and process rate determinations.

Compounds added (5µM unless otherwise stated)	Processes investigated
$^{15}N\text{-}NH_4^+$, $^{14}N\text{-}NO_2^-$, $^{14}N\text{-}NO_3^-$, ^{13}C-DIC *	Ammonium assimilation, and carbon fixation
$^{15}N^{13}C$-Urea, $^{14}N\text{-}NO_2^-$, $^{14}N\text{-}NO_3^-$	Urea assimilation, and carbon fixation
$^{15}N^{13}C$-Cyanate, $^{14}N\text{-}NO_2^-$, $^{14}N\text{-}NO_3^-$	Cyanate assimilation, and carbon fixation
$^{15}N\text{-}NO_2^-$, $^{14}N\text{-}NO_3^-$,^{13}C-DIC *	Nitrite assimilation and oxidation, and carbon fixation

^{15}N-ammonium sulfate (98% ^{15}N, Sigma), $^{15}N^{13}C$-urea (99% ^{13}C, 98% ^{15}N, Sigma), $^{15}N^{13}C$-potassium cyanate (95% purity, 99% ^{13}C, 98% ^{15}N, Icon Isotopes), ^{13}C-sodium bicarbonate (98% ^{13}C, Sigma), ^{14}N-compounds were all obtained from Sigma
* 200 µM final $^{13}C\text{-}NaHCO_3$ concentration

Supplementary Table 5 Sequencing statistics for GoM metagenomes.

Station, Depth	Dataset	# Raw reads	# Raw fragments (read pairs)	# Raw bases	# Total QC fragments (read pairs, merged reads and unpaired reads)	# Total QC bases	# Scaffolds	Scaffold length (MB)	Scaffold N50	Scaffold L50 (KB)	Max scaffold length (KB)
Station 1, 18.0 m	C6-18m_S5	72,032,256	36,016,128	17,957,359,367	34,754,578	16,103,062,180	923,189	1,332	172,469	1.556	408
Station 2, 12.0 m	I3-11-9m_S1	84,917,344	42,458,672	21,090,538,252	41,340,486	17,634,270,666	978,822	1,352	189,985	1.46	377
Station 2, 14.0 m	I3-13-9m_S3	58,352,508	29,176,254	14,547,679,676	28,095,278	12,983,602,885	636,633	957	113,296	1.695	746
Station 2, 16.5 m	I3-16-4m_S2	104,108,154	52,054,077	25,988,959,289	50,284,474	23,548,567,999	964,193	1,545	148,342	1.921	950
Station 3, 14.0 m	CPI-14m_S4	93,064,228	46,532,114	23,218,119,059	44,629,516	20,762,427,575	931,744	1,407	162,385	1.719	958

Supplementary Table 6 Metatranscriptome sequencing statistics.

Station, Depth	Dataset	# Raw reads	# Raw fragments (read pairs)	# Raw bases	# rRNA-free fragments	# rRNA-free bases
Station 2, 12.0 m	SRR5909422	9,359,372	4,679,686	2,468,604,609	1,843,080	1,535,588,485
Station 2, 14.0 m	SRR5909421	19,415,124	9,707,562	3,505,604,022	7,241,278	2,599,084,484
Station 2, 16.5 m	SRR5909415	18,976,144	9,488,072	3,265,534,600	6,557,865	2,243,101,279

Supplementary Table 7 Phylogenetic affiliation of Nitrospinae (target population) and non-Nitrospinae (non-target population) 16S rRNA gene sequences with perfect match to newly designed probe Ntspn759, and previously published probes Ntspn693 [1] and Ntspn-Mod [2] in Silva_132_SSURef_NR99_13_12_17.

	**Phylum**	**Match/total**			**Class / Order / Family**	**Match/total**			**Genus**	**Match/total**		
		**Ntspn759**	**Ntspn-Mod**	**Ntspn693**		**Ntspn759**	**Ntspn-Mod**	**Ntspn693**		**Ntspn759**	**Ntspn-Mod**	**Ntspn693**
Target	Nitrospinae	263/364	228/364	43/364	Nitrospinia/ Nitrospinales/ Nitrospinaceae	263/288	228/288	43/288	Genus *Nitrospina*	150/171	128/171	31/171
									LS-NOB (Clade 2)	100/103	98/103	0/103
									uncultured	13/14	2/14	12/14
Non-Target	Spirochaetes	1/4,253			Spirochaetia	1/3,637						
	WOR-1	1/34										
	Planctomycetes	22/9,014			Planctomycetacia	15/5,149						
					Phycisphaerae	7/2,568						
	Marinimicrobia (SAR406 clade)	1/554										
	Chloroflexi		2/9,245	21/9,245	Dehalococcoidia		2/1,606	21/1,606				
	Patescibacteria			1/4,521	WS6 (Dojkabacteria)			1/162				

Supplementary Figures

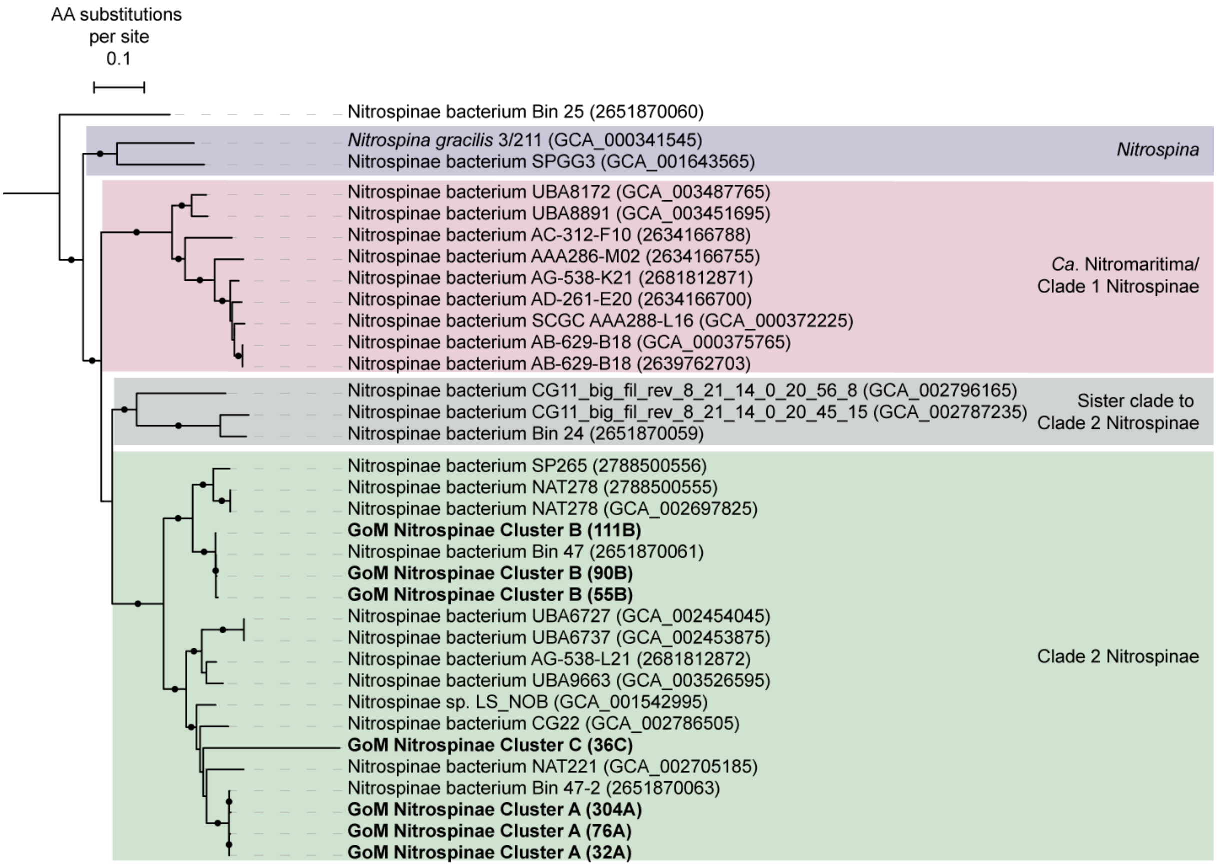

Supplementary Figure 1 Nitrospinae genome tree based on 34 universal concatenated marker genes. Nitrospinae GoM metagenome assembled genomes (GoM Nitrospinae) are printed in bold. Deltaproteobacterial genomes were used as an outgroup. Scale bar represents estimated amino acid substitutions per site, and bootstrap values > 90% are displayed.

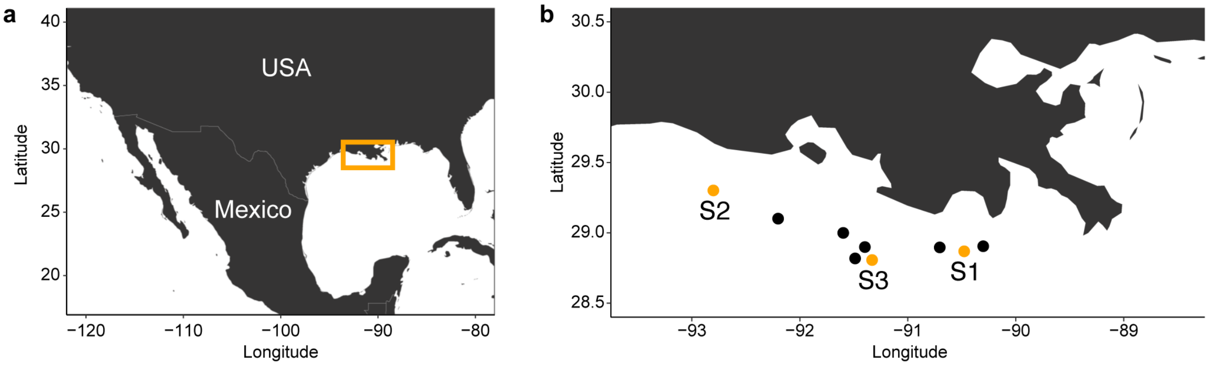

Supplementary Figure 2 Sampling transect in the GoM. a) The GoM sampling location is marked by an orange square. b) Station locations; experimental stations are indicated with orange dots (S1 – S3), stations for nutrient and CTD profiles by black dots. Station map was produced in R[23] using the "maps" package and has been modified from Kitzinger et al.[12].

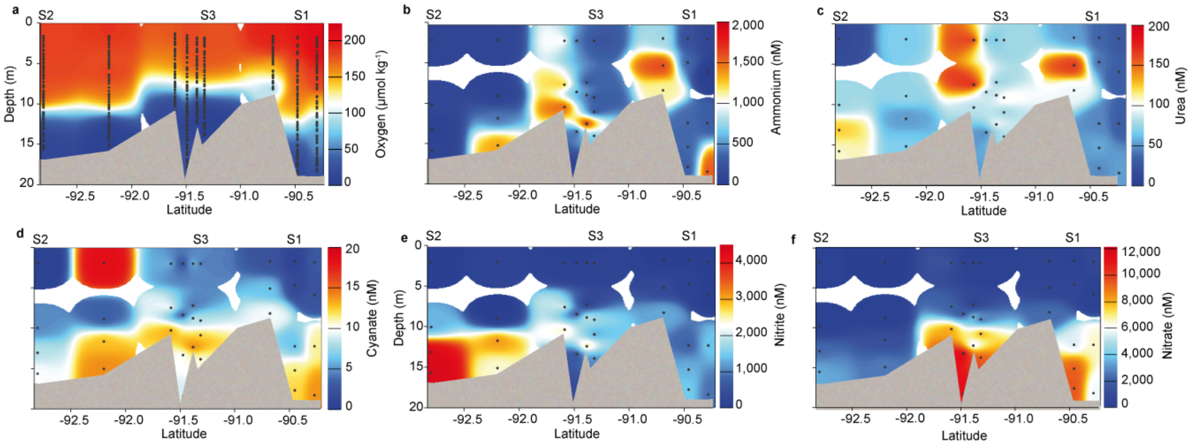

Supplementary Figure 3 Oxygen and nutrient profiles across the sampling transect (shaded area indicates the seafloor). a) Oxygen, b) ammonium, c) urea, d) cyanate, e) nitrite, f) nitrate concentrations. Black dots mark locations of measurements. S1 – S3 mark the position of experimental stations. Plots were generated using Ocean Data View[45]. Concentration profiles have been previously published by Kitzinger et al.[12]

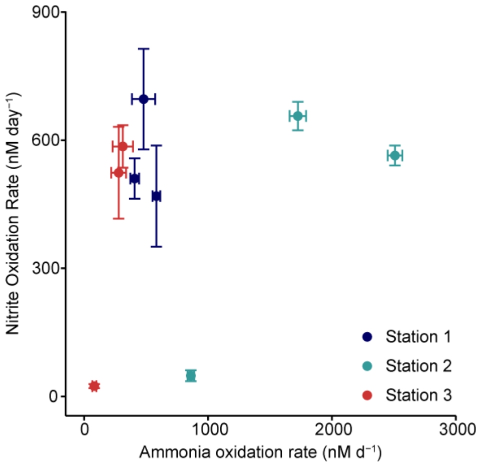

Supplementary Figure 4 Correlation between measured ammonia and nitrite oxidation rates in the GoM. Nitrite and ammonia oxidation rates were calculated from slopes across all time points of triplicate incubations. Error bars represent standard error of the slope.

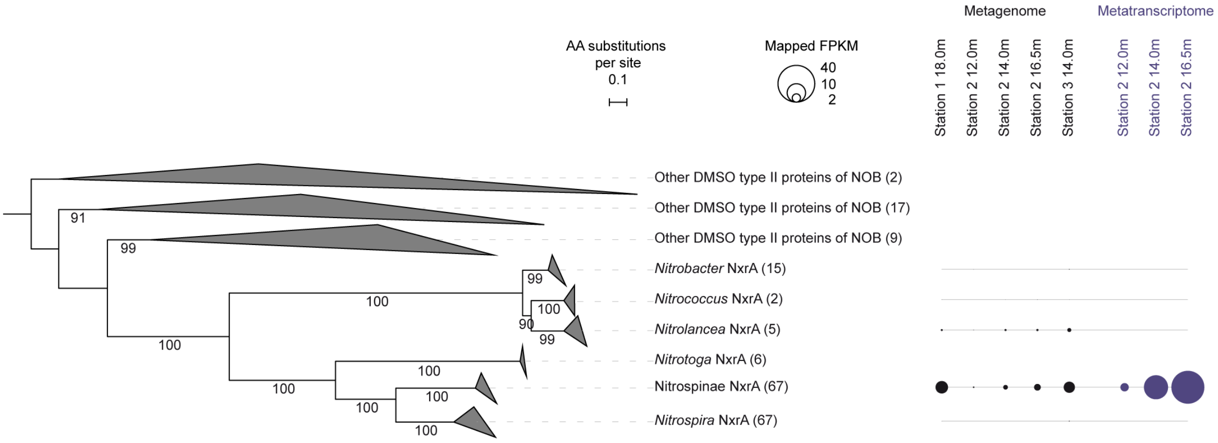

Supplementary Figure 5 NOB-affiliated NxrA and additional NOB-affiliated DMSO reductase family type II enzyme amino acid sequences retrieved from GoM metagenomes and metatranscriptomes. Outgroup are Molybdopterin oxidoreductase-like genes of *Nitrobacter* and *Nitrolancea*. GoM metagenomic and metatranscriptomic read fragments (FPKM) were mapped onto the alignment and are shown next to the respective clades as circles. The scale bar represents estimated amino acid substitutions per site, and bootstrap values > 90% are displayed. Accession numbers of reference sequences included are given in Supplementary Data 2.

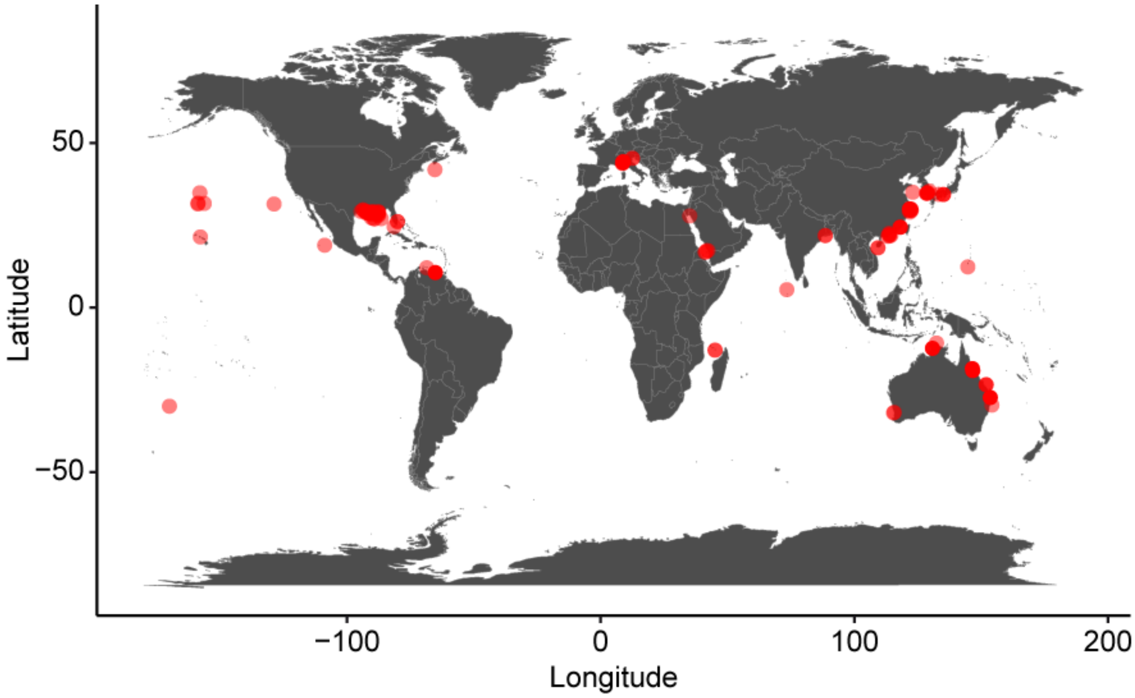

Supplementary Figure 6 Distribution of 16S rRNA gene phylotypes in amplicon datasets closely related (>99% identity) to full length GoM Nitrospinae Clade 2 16S rRNA gene sequences obtained from GoM metagenomes. Red transparent circles indicate the presence of GoM Nitrospinae Clade 2-related sequences across all publicly available 16S rRNA gene amplicon datasets. Overlapping circles due to presence of target sequences in multiple datasets obtained from one location (or multiple locations in close vicinity) appear darker. Figure was produced in R using the "maps" package[23].

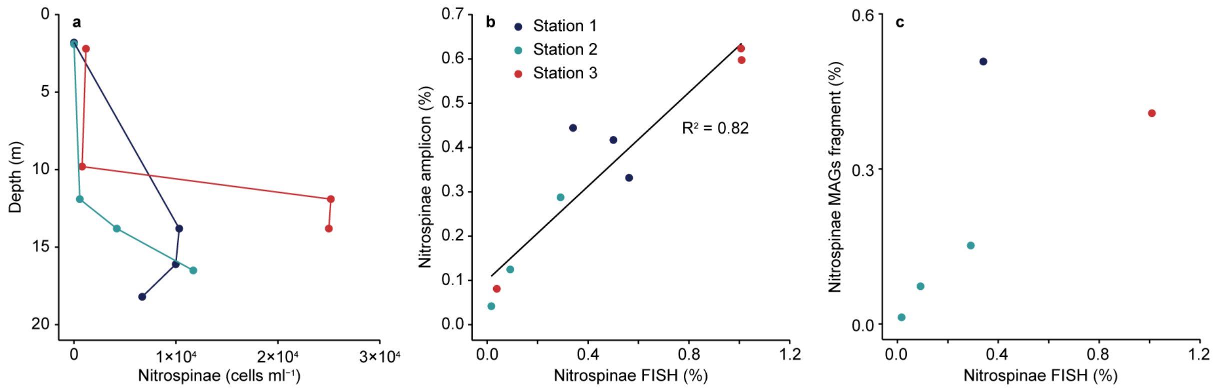

Supplementary Figure 7 Nitrospinae cell abundance based on CARD-FISH (a) and correlation to Nitrospinae relative abundance from 16S rRNA gene amplicon sequencing (b) and Nitrospinae MAG abundance in the metagenomic datasets, based on mapping of metagenomic read fragments to Nitrospinae MAG population clusters A, B and C (c). The black line represents linear regression ($p = 0.0002$), R^2 was calculated based on Pearson Correlation.

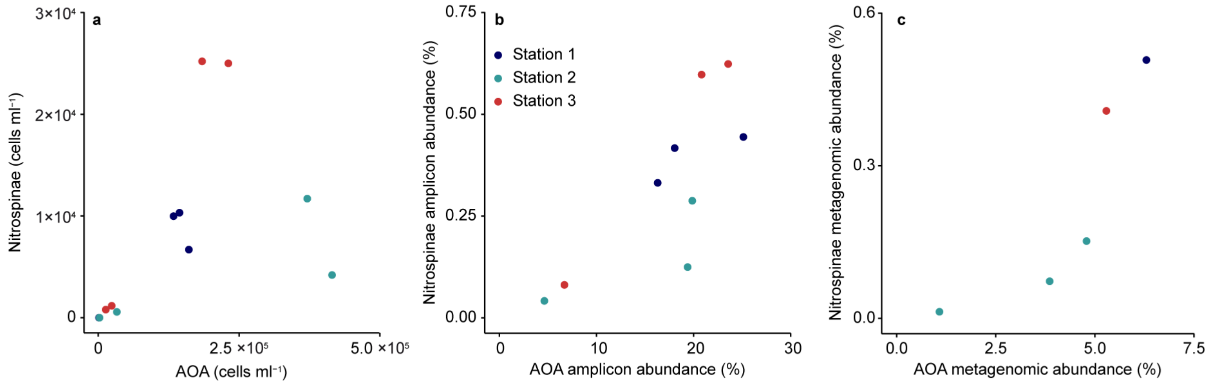

Supplementary Figure 8 Nitrospinae and AOA cell abundance based on CARD-FISH (a), relative abundance from 16S rRNA gene amplicon sequencing b) and MAGs c). Note the different scaling of axes for Nitrospinae and AOA.

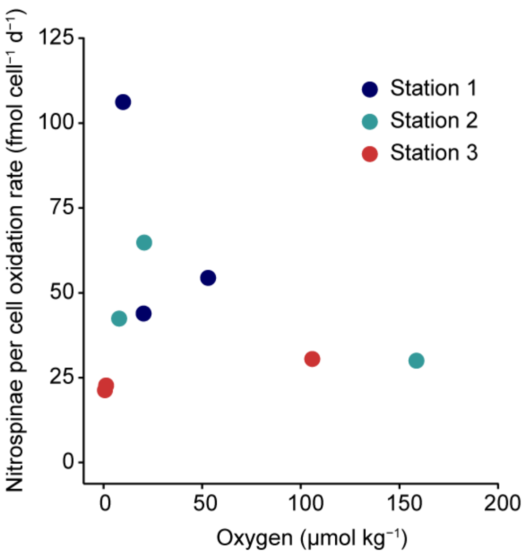

Supplementary Figure 9 Correlation of Nitrospinae per cell nitrite oxidation rates and *in situ* oxygen concentration in the GoM.

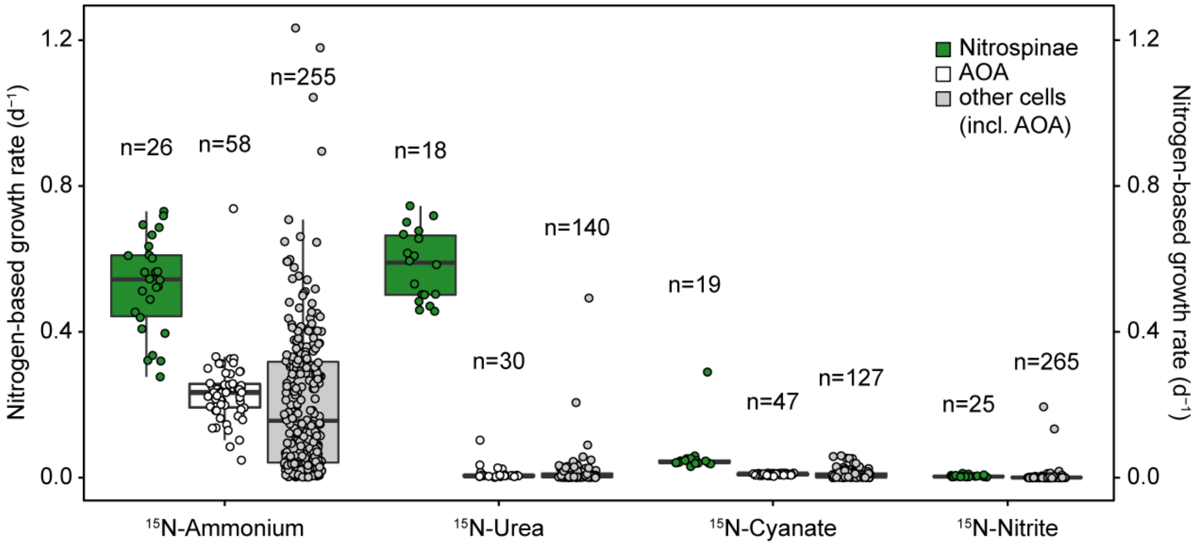

Supplementary Figure 10 Nitrospinae single cell growth rates measured by nanoSIMS. Nitrospinae are depicted in green, AOA in white and other, non-targeted cells in grey. Growth rates were calculated from single cell ^{15}N-enrichment after incubation with ^{15}N-ammonium, ^{15}N-urea, ^{15}N-cyanate or ^{15}N-nitrite. AOA data was taken from Kitzinger et al.[12] for comparison. Note that non-targeted cells depicted here also include AOA cells, as no specific AOA probe was included in the Nitrospinae experiments. Number of cells analyzed per category is indicated above each boxplot. Boxplots depict the 25 – 75 % quantile range, with the center line depicting the median (50% quantile); whiskers encompass data points within 1.5 × the interquartile range.

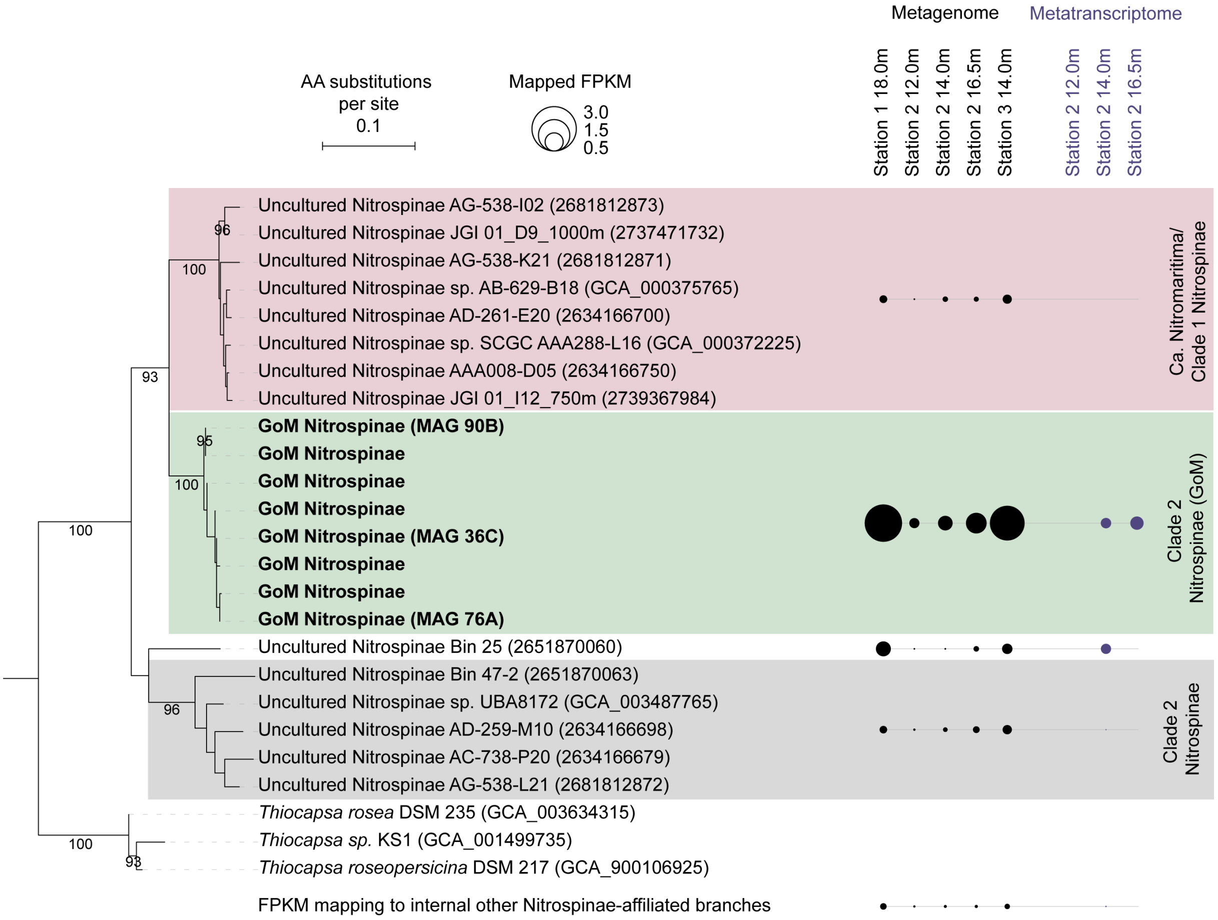

Supplementary Figure 11 Nitrospinae-affiliated UreC amino acid sequences retrieved from GoM metagenomes and metatranscriptomes (for full UreC diversity retrieved from GoM metagenomes and metatranscriptomes refer to Kitzinger et al.[12]). Metagenome assembled Nitrospinae-affiliated UreC sequences are indicated as "GoM Nitrospinae" and printed in bold. Outgroup are *Thiocapsa*-affiliated UreC sequences. GoM metagenomic and metatranscriptomics read fragments (FPKM) were mapped onto a subset of the alignment excluding the *Thiocapsa* outgroup and are shown next to the respective clades as circles. FPKM mapping to internal basal nodes were grouped and are displayed separately. The scale bar represents estimated amino acid substitutions per site, and bootstrap values > 90% are displayed. Accession numbers of reference sequences included are given in Supplementary Data 2.

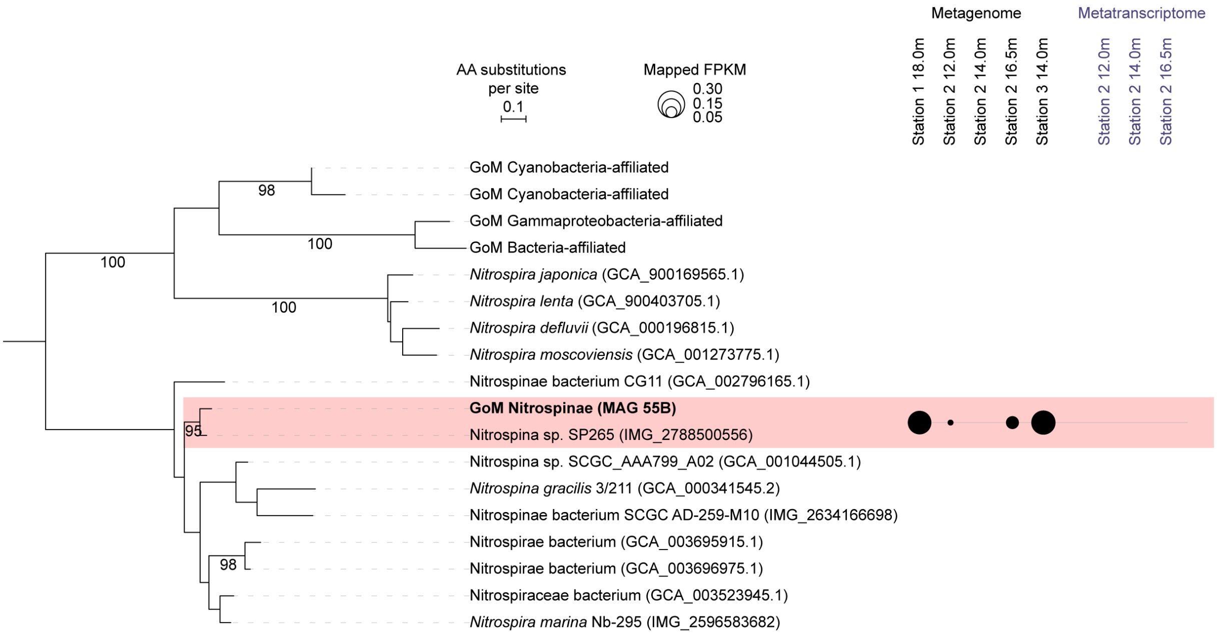

Supplementary Figure 12 Subset of CynS amino acid sequences retrieved from GoM metagenomes and metatranscriptomes (for full CynS diversity retrieved from GoM metagenomes and metatranscriptomes refer to Kitzinger et al.[12]). Metagenome assembled Nitrospinae-affiliated CynS sequences are indicated as “GoM Nitrospinae” and printed in bold. Outgroup are terrestrial *Nitrospira* and GoM non-Nitrospinae-affiliated CynS sequences. GoM metagenomic and metatranscriptomics read fragments (FPKM) were mapped onto a subset of the alignment including Nitrospinae and marine *Nitrospira* sequences and are shown next to the respective clades as circles. FPKM mapping to internal basal nodes were grouped and are displayed separately. The scale bar represents estimated amino acid substitutions per site, and bootstrap values > 90% are displayed. Accession numbers of reference sequences included are given in Supplementary Data 2.

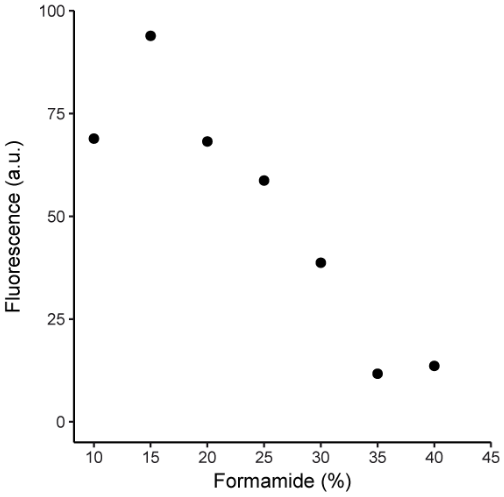

Supplementary Figure 13 Formamide concentration series for probe Ntspn759. CARD-FISH was performed using different formamide concentrations on a PFA-fixed pure culture of *N. gracilis*. Fluorescence of hybridized cells was recorded under identical conditions for all tested formamide concentrations. Fluorescence intensity dropped at formamide concentrations >20%. a.u., artificial units of fluorescence intensity.

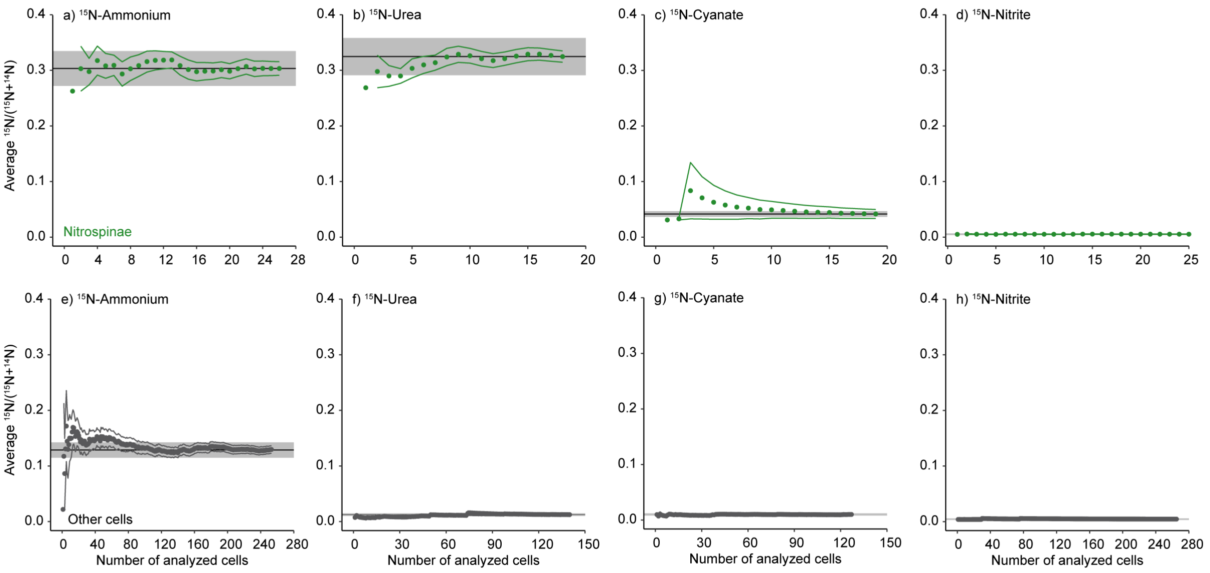

Supplementary Figure 14 Enrichment statistics of Nitrospinae cells (green) and other cells (dark grey) analyzed by nanoSIMS. Dots and solid lines represent the means and standard errors, respectively, of $^{15}N/(^{15}N+^{14}N)$ ratios calculated across randomly subsampled cells. The black line represents the mean across all cells, the light grey area ± 10% of the mean. Standard errors for Nitrospinae were < 10% after analysis of 3, 2 and 3 cells in the ^{15}N-ammonium, ^{15}N-urea and ^{15}N-nitrite treatments, for the ^{15}N-cyanate treatment, one Nitrospinae cell with higher activity caused the error to remain 19% after analysis of all 19 Nitrospinae cells. For other, non-Nitrospinae cells (e-h), the standard error of $^{15}N/(^{14}N+^{15}N)$ values was <10% after measurement of 74, 60, and 185 cells in the ^{15}N-ammonium, ^{15}N-cyanate and ^{15}N-nitrite treatments. For ^{15}N-urea, the standard error did not drop < 10% after analysis of 140 non-Nitrospinae cells. The total number of analyzed cells was a) n = 26, b) n=18, c) n=19, d) n=25, e) n=255, f) n=140, g) n=127, h) n=265.

Supplementary References

1. Juretschko, S. Mikrobielle Populationsstruktur und --dynamik in einer nitrifizierenden/denitrifizierenden Belebtschlammanlage. (Technische Universität München, 2000).
2. Pachiadaki, M. G. *et al.* Major role of nitrite-oxidizing bacteria in dark ocean carbon fixation. *Science (80-.).* **358**, 1046–1051 (2017).
3. Yilmaz, P. *et al.* The SILVA and 'all-species Living Tree Project (LTP)' taxonomic frameworks. *Nucleic Acids Res.* **42**, D643–D648 (2014).
4. Ludwig, W. *et al.* ARB: A software environment for sequence data. *Nucleic Acids Res.* **32**, 1363–1371 (2004).
5. Caporaso, J. G. *et al.* Global patterns of 16S rRNA diversity at a depth of millions of sequences per sample. *Proc. Natl. Acad. Sci. U. S. A.* **108**, 4516–4522 (2011).
6. Yilmaz, L. S., Parnerkar, S. & Noguera, D. R. MathFISH, a web tool that uses thermodynamics-based mathematical models for in silico evaluation of oligonucleotide probes for fluorescence in situ hybridization. *Appl. Environ. Microbiol.* **77**, 1118–1122 (2011).
7. Manz, W., Amann, R., Ludwig, W., Wagner, M. & Schleifer, K. H. Phylogenetic oligodeoxynucleotide probes for the major subclasses of Proteobacteria: problems and solutions. *Syst. Appl. Microbiol.* **15**, 593–600 (1992).
8. Watson, S. W. & Waterbury, J. B. Characteristics of Two Marine Nitrite Oxidizing Bacteria, Nitrospina gracilis nov. gen. nov. sp. and Nitrococcus mobilis nov. gen. nov. sp. *Arch. Microbiol.* **77**, 203–230 (1971).
9. Pernthaler, A., Pernthaler, J. & Amann, R. Sensitive multi-color fluorescence in situ hybridization for the identification of environmental microorganisms. in *Molecular Microbial Ecology Manual* (eds. Kowalchuk, G., de Bruijn, F. J., Head, I. M., Akkermans, A. D. L. & van Elsas, J. D.) 711–726 (Kluwer Academic Publishers, 2004). doi:10.1007/978-1-4020-2177-0_311
10. Wallner, G., Amann, R. & Beisker, W. Optimizing fluorescent in situ hybridization with rRNA-targeted oligonucleotide probes for flow cytometric identification of microorganisms. *Cytometry* **14**, 136–143 (1993).
11. Svedén, J. B. *et al.* High cell-specific rates of nitrogen and carbon fixation by the cyanobacterium

Aphanizomenon sp. at low temperatures in the Baltic Sea. *FEMS Microbiol. Ecol.* **91**, 1–10 (2015).

12. Kitzinger, K. *et al.* Cyanate and urea are substrates for nitrification by Thaumarchaeota in the marine environment. *Nat. Microbiol.* **4**, 234–243 (2019).

13. Padilla, C. C. *et al.* NC10 bacteria in marine oxygen minimum zones. *ISME J.* **10**, 2067–2071 (2016).

14. Frias-Lopez, J. *et al.* Microbial community gene expression in ocean surface waters. *Proc. Natl. Acad. Sci. U. S. A.* **105**, 3805–3810 (2008).

15. Magoč, T. & Salzberg, S. L. FLASH: Fast length adjustment of short reads to improve genome assemblies. *Bioinformatics* **27**, 2957–2963 (2011).

16. Edgar, R. C. Search and clustering orders of magnitude faster than BLAST. *Bioinformatics* **26**, 2460–2461 (2010).

17. Caporaso, J. G. *et al.* QIIME allows analysis of high-throughput community sequencing data. *Nat. Methods* **7**, 335–336 (2010).

18. DeSantis, T. Z. *et al.* Greengenes, a chimera-checked 16S rRNA gene database and workbench compatible with ARB. *Appl. Environ. Microbiol.* **72**, 5069–5072 (2006).

19. Nurk, S., Meleshko, D., Korobeynikov, A. & Pevzner, P. A. MetaSPAdes: A new versatile metagenomic assembler. *Genome Res.* **27**, 824–834 (2017).

20. Kang, D. D., Froula, J., Egan, R. & Wang, Z. MetaBAT, an efficient tool for accurately reconstructing single genomes from complex microbial communities. *PeerJ* **3**, e1165 (2015).

21. Olm, M. R., Brown, C. T., Brooks, B. & Banfield, J. F. DRep: A tool for fast and accurate genomic comparisons that enables improved genome recovery from metagenomes through de-replication. *ISME J.* **11**, 2864–2868 (2017).

22. Varghese, N. J. *et al.* Microbial species delineation using whole genome sequences. *Nucleic Acids Res.* **43**, 6761–6771 (2015).

23. R Development Core Team. R: A language and environment for statistical computing. *R Foundation for Statistical Computing* (2013). doi:10.1017/CBO9781107415324.004

24. Parks, D. H. *et al.* A standardized bacterial taxonomy based on genome phylogeny substantially revises the tree of life. *Nat. Biotechnol.* **36**, 996–1004 (2018).

25. Dyksma, S. *et al.* Ubiquitous Gammaproteobacteria dominate dark carbon fixation in coastal sediments.

ISME J. **10**, 1939–1953 (2016).

26. Mußmann, M., Pjevac, P., Krüger, K. & Dyksma, S. Genomic repertoire of the Woeseiaceae/JTB255, cosmopolitan and abundant core members of microbial communities in marine sediments. *ISME J.* **11**, 1276–1281 (2017).

27. Pjevac, P. *et al.* In situ abundance and carbon fixation activity of distinct anoxygenic phototrophs in the stratified seawater lake Rogoznica . *Environ. Microbiol.* **21**, 3896–3908 (2019).

28. Bankevich, A. *et al.* SPAdes: a new genome assembly algorithm and its applications to single-cell sequencing. *J. Comput. Biol.* **19**, 455–77 (2012).

29. Wheeler, T. J. & Eddy, S. R. Nhmmer: DNA homology search with profile HMMs. *Bioinformatics* **29**, 2487–2489 (2013).

30. Kalvari, I. *et al.* Rfam 13.0: Shifting to a genome-centric resource for non-coding RNA families. *Nucleic Acids Res.* **46**, D335–D342 (2018).

31. Wang, Q., Garrity, G. M., Tiedje, J. M. & Cole, J. R. Naïve Bayesian classifier for rapid assignment of rRNA sequences into the new bacterial taxonomy. *Appl. Environ. Microbiol.* **73**, 5261–5267 (2007).

32. Schloss, P. D. *et al.* Introducing mothur: Open-source, platform-independent, community-supported software for describing and comparing microbial communities. *Appl. Environ. Microbiol.* **75**, 7537–7541 (2009).

33. Hyatt, D. *et al.* Prodigal: prokaryotic gene recognition and translation initiation site identification. *BMC Bioinformatics* **11**, 1–11 (2010).

34. Eddy, S. R. Accelerated profile HMM searches. *PLoS Comput. Biol.* **7**, e1002195 (2011).

35. Karsch-Mizrachi, I., Takagi, T. & Cochrane, G. The international nucleotide sequence database collaboration. *Nucleic Acids Res.* **46**, D48–D51 (2018).

36. Chen, I. M. A. *et al.* IMG/M v.5.0: An integrated data management and comparative analysis system for microbial genomes and microbiomes. *Nucleic Acids Res.* **47**, D666–D677 (2019).

37. Katoh, K., Misawa, K., Kuma, K. & Miyata, T. MAFFT: a novel method for rapid multiple sequence alignment based on fast Fourier transform. *Nucleic Acids Res.* **30**, 3059–3066 (2002).

38. Capella-Gutiérrez, S., Silla-Martínez, J. M. & Gabaldón, T. trimAl: A tool for automated alignment trimming in large-scale phylogenetic analyses. *Bioinformatics* **25**, 1972–1973 (2009).

39. Nguyen, L. T., Schmidt, H. A., Von Haeseler, A. & Minh, B. Q. IQ-TREE: A fast and effective stochastic algorithm for estimating maximum-likelihood phylogenies. *Mol. Biol. Evol.* **32**, 268–274 (2015).

40. Kalyaanamoorthy, S., Minh, B. Q., Wong, T. K. F., Von Haeseler, A. & Jermiin, L. S. ModelFinder: Fast model selection for accurate phylogenetic estimates. *Nat. Methods* **14**, 587–589 (2017).

41. Hoang, D. T., Chernomor, O., von Haeseler, A., Minh, B. Q. & Vinh, L. S. UFBoot2: Improving the Ultrafast Bootstrap Approximation. Molecular biology and evolution. *Mol. Biol. Evol.* **35**, 518–522 (2017).

42. Letunic, I. & Bork, P. Interactive tree of life (iTOL) v3: an online tool for the display and annotation of phylogenetic and other trees. *Nucleic Acids Res.* **44**, W242–W245 (2016).

43. Berger, S. A., Krompass, D. & Stamatakis, A. Performance, accuracy, and web server for evolutionary placement of short sequence reads under maximum likelihood. *Syst. Biol.* **60**, 291–302 (2011).

44. Beman, J. M. *et al.* Global declines in oceanic nitrification rates as a consequence of ocean acidification. *Proc. Natl. Acad. Sci. U. S. A.* **108**, 208–213 (2011).

45. Schlitzer, R. Ocean Data View. (2016).
