# Supplementary material for: Single cell analyses reveal contrasting life strategies of the two main nitrifiers in the ocean
Source: Nat Commun. 2020 Feb 7;11:767. doi: 10.1038/s41467-020-14542-3 (PMC7005884; doi:10.1038/s41467-020-14542-3)
Supplement: Supplementary file 5 — Reporting Summary [file 41467_2020_14542_MOESM5_ESM.pdf]

nature research

Corresponding author(s): Katharina Kitzinger and Hannah K. Marchant

Last updated by author(s): Jan 7, 2020

Reporting Summary

Nature Research wishes to improve the reproducibility of the work that we publish. This form provides structure for consistency and transparency in reporting. For further information on Nature Research policies, see Authors & Referees and the Editorial Policy Checklist .

Statistics

For all statistical analyses, confirm that the following items are present in the figure legend, table legend, main text, or Methods section.

n/a	Confirmed	
☐	☒	The exact sample size (*n*) for each experimental group/condition, given as a discrete number and unit of measurement
☐	☒	A statement on whether measurements were taken from distinct samples or whether the same sample was measured repeatedly
☐	☒	The statistical test(s) used AND whether they are one- or two-sided<br>*Only common tests should be described solely by name; describe more complex techniques in the Methods section.*
☒	☐	A description of all covariates tested
☒	☐	A description of any assumptions or corrections, such as tests of normality and adjustment for multiple comparisons
☐	☒	A full description of the statistical parameters including central tendency (e.g. means) or other basic estimates (e.g. regression coefficient) AND variation (e.g. standard deviation) or associated estimates of uncertainty (e.g. confidence intervals)
☐	☒	For null hypothesis testing, the test statistic (e.g. *F*, *t*, *r*) with confidence intervals, effect sizes, degrees of freedom and *P* value noted<br>*Give P values as exact values whenever suitable.*
☒	☐	For Bayesian analysis, information on the choice of priors and Markov chain Monte Carlo settings
☒	☐	For hierarchical and complex designs, identification of the appropriate level for tests and full reporting of outcomes
☐	☒	Estimates of effect sizes (e.g. Cohen's *d*, Pearson's *r*), indicating how they were calculated

Our web collection on statistics for biologists contains articles on many of the points above.

Software and code

Policy information about availability of computer code

Data collection	No software was used for data collection.
Data analysis	Microsoft Excel, R v. 3.5.1., arb-silva test-prime, Arb v. 6.1., MathFISH, Trim Galore!, FLASH, USEARCH, UCLUST, QIME1, MUSCLE, RAxML v. 8.2.11, EPA (RAxML v. 8.2.11), FigTree v1.4.3, Metaspades v. 3.10.1., SPAdes v. 3.10.1., BBduk, BBMap v. 36.32, Metabat2 v. 2.12.1., GTDB-Tk v. 0.2.2., dRep v. 1.4.3., FastTree2, CheckM v. 1.0.7., CD-HIT, IQ-TREE v. 1.6.2., ModelFinder, ITOL, RDPclassifier (Mothur v. 1.39.5), Prodigal, hmmer v. 3.1b2, Integrated Microbial Genomes & Microbiomes system v. 5.0, mafft-linsi v. 7.397, trimal v. 1.4.1, ITOL 4.2.3, MAFFT v. 7.397, SortMeRNA v. 2.1., Look@NanoSIMS 2015-06-09

For manuscripts utilizing custom algorithms or software that are central to the research but not yet described in published literature, software must be made available to editors/reviewers. We strongly encourage code deposition in a community repository (e.g. GitHub). See the Nature Research guidelines for submitting code & software for further information.

Data

Policy information about availability of data

All manuscripts must include a data availability statement. This statement should provide the following information, where applicable:

- Accession codes, unique identifiers, or web links for publicly available datasets
- A list of figures that have associated raw data
- A description of any restrictions on data availability

All sequence data and Nitrospinae MAGs generated in this study are deposited in NCBI under BioProject number: PRJNA397176. Metatranscriptomes are deposited under BioSample numbers SAMN07461123–SAMN07461125; 16S amplicon sequencing under SAMN07461114–SAMN07461122; metagenomes under SAMN10227777–SAMN10227781 and MAGs under SAMN12766710–SAMN12766716. Accession numbers of sequences used for tree calculations (16S rRNA gene, NxrA, UreC, CynS, and genome sequences) are given in Supplementary Data 2. CTD data, measured nutrient concentrations, process rates, Nitrospinae and AOA relative abundance based on 16S rRNA gene amplicon sequencing and CARD-FISH counts are given in Supplementary Data 1.

Field-specific reporting

Please select the one below that is the best fit for your research. If you are not sure, read the appropriate sections before making your selection.

☒ Life sciences ☐ Behavioural & social sciences ☐ Ecological, evolutionary & environmental sciences

For a reference copy of the document with all sections, see nature.com/documents/nr-reporting-summary-flat.pdf

Life sciences study design

All studies must disclose on these points even when the disclosure is negative.

Sample size	No statistical methods were used to pre-determine sample size. Field experiments in the Gulf of Mexico were done with three independent biological replicates (water samples), which is standard procedure for environmental biogeochemical rate measurements. For nanoSIMS analyses, we performed statistical tests to show that sufficient cells have been measured. Please refer also to the methods section.
Data exclusions	No data were excluded from analysis
Replication	All attempts at replication were successful. Similar results were obtained for all biological replicates. Data was analyzed by three people independently.
Randomization	Biological triplicates per treatment were considered as one experimental group, as rates were calculated across biological triplicates and all time points for environmental rates.
Blinding	Blinding was not applicable because the study does not involve animals and/or human research participants.

Reporting for specific materials, systems and methods

We require information from authors about some types of materials, experimental systems and methods used in many studies. Here, indicate whether each material, system or method listed is relevant to your study. If you are not sure if a list item applies to your research, read the appropriate section before selecting a response.

Materials & experimental systems

n/a	Involved in the study	
☒	☐	Antibodies
☒	☐	Eukaryotic cell lines
☒	☐	Palaeontology
☒	☐	Animals and other organisms
☒	☐	Human research participants
☒	☐	Clinical data

Methods

n/a	Involved in the study	
☒	☐	ChIP-seq
☒	☐	Flow cytometry
☒	☐	MRI-based neuroimaging
